# Antifungal Activity of Silver Salts of Keggin-Type Heteropolyacids Against *Sporothrix *spp.

**DOI:** 10.4014/jmb.1907.07064

**Published:** 2019-12-30

**Authors:** Luciana Da Silva Mathias, João Carlos De Aquino Almeida, Luis César Passoni, Cristiani Miranda David Gossani, Gabriel Bonan Taveira, Valdirene Moreira Gomes, Olney Vieira-Da-Motta

**Affiliations:** 1 Laboratório de Sanidade Animal, Hospital Veterinário, Centro de Ciências e Tecnologias Agropecuárias, Universidade Estadual do Norte Fluminense Darcy Ribeiro, 28013-602, Campos dos Goytacazes, Rio de Janeiro, Brazil; 2 Laboratório de Fisiologia e Bioquímica de Microrganismos, Centro de Biociências e Biotecnologia, Universidade Estadual do Norte Fluminense Darcy Ribeiro, 28013-602, Campos dos Goytacazes, Rio de Janeiro, Brazil; 3 Laboratório de Ciências Químicas, Centro de Ciências e Tecnologias, Universidade Estadual do Norte Fluminense Darcy Ribeiro, 28013-602, Campos dos Goytacazes, Rio de Janeiro, Brazil

**Keywords:** Pathogenic fungus, zoonosis, silver, polyoxometalate, antifungal effect

## Abstract

Sporotrichosis is a chronic and subacute mycosis causing epidemiological outbreaks involving sick cats and humans in southeastern Brazil. The systemic disease prevails in cats and in humans, with the symptoms restricted to the skin of immunocompetent individuals. Under these conditions, the prolonged treatment of animals and cases of recurrence justify the discovery of new treatments for sporotrichosis. This work addresses the antifungal activity of silver salts of Keggin-type heteropolyacid salts (Ag-HPA salts) such as Ag_3_[PW_12_O_40_], Ag_6_[SiW_10_V_2_O_40_], Ag_4_[SiW_12_O_40_] and Ag_3_[PMo_12_O_40_] and interactions with the antifungal drugs itraconazole (ITC), terbinafine (TBF) and amphotericin B (AMB) on the yeast and mycelia forms of *Sporothrix* spp. *Sporothrix* spp. yeast cells were susceptible to Ag-HPA salts at minimum inhibitory concentration (MIC) values ranging from 8 to 128 μg/ml. Interactions between Ag_3_[PW_12_O_40_] and Ag_3_[PMo_12_O_40_] with itraconazole and amphotericin B resulted in higher antifungal activity with a reduction in growth and melanization. Treated cells showed changes in cell membrane integrity, vacuolization, cytoplasm disorder, and membrane detachment. Promising antifungal activity for treating sporotrichosis was observed for the Ag-HPA salts Ag_3_[PMo_12_O_40_] and Ag_3_[PW_12_O_40_], which have a low cost, high yield and activity at low concentrations. However, further evaluation of in vivo tests is still required.

## Introduction

*Sporothrix* spp. are dimorphic, saprophytic and geophilic fungi of the *Sporothrix* complex and responsible for sporotrichosis, a chronic infection of the skin and subcutaneous tissue of humans and animals [1]. Although they are distributed worldwide, the frequency of these fungi in tropical regions is more common due to the warm and moist climate [1, 2]. In Brazil, three states are among the most affected, São Paulo, Rio Grande do Sul, and Rio de Janeiro, where human sporotrichosis is considered an epidemic re-emerging disease, especially in underserved populations and immunocompromised patients [3, 4]. The classic route of transmission of *Sporothrix* spp. involves traumatic cutaneous inoculation of the fungus by contact with infected cats or contaminated environmental sources [5]. The usual treatment for sporotrichosis is based on potassium iodine and itraconazole, although treating sick cats to control this epidemic has been a challenge, and amphotericin B is used for refractory or systemic cases [6-9]. In this context, the occurrence of side effects due to prolonged treatment, especially on felines, has driven the development of alternative drugs to treat this disease.

Polyoxometalates (POMs) are nanosized structures with potential chemical applications in catalysis, materials science, magnetism and medicine [10]. The structures of POMs may or may not contain heteroatoms, which divides this class of compounds into two groups: (1) the isopolianions of the formula (MmOy)^x-^ and (2) heteropolyanions of the formula (XnMmOy)^x-^ [11]. Biologically, POMs inhibit the replication of various nonretro RNA and DNA viruses and tumor cells and act synergistically when combined with antibiotics against methicillin and vancomycin-resistant *Staphylococcus aureus* [12, 13]. Keggin-type heteropolyacids (HPAs) are molecules formed by a central tetrahedron XO_4_, where X may be chosen among any element with the ability for tetrahedral coordination, and usually silicon (Si) or phosphorus (P) are the preferred choice [13]. Several Keggin-type HPA salts containing the cations Na^+^, Fe^3+^, K^+^, Ce^3+^, and Ba^2+^ showed activity against viruses and bacteria [15].

Silver ion metal (Ag^+^) and silver nanoparticles (AgNPs) have been known for centuries as highly toxic to microorganisms, showing strong antimicrobial effects against fungi and bacteria [16-18]. The positive charge of silver ions is crucial for their antimicrobial activity through electrostatic attraction toward the negatively charged cell membrane of microorganisms, causing cell lysis [19]. In *Candida albicans*, AgNPs break down the membrane permeability barrier by perturbing membrane lipid bilayers, causing leakage of ions and other materials as well as forming pores and dissipating the electrical potential of the membrane [18].

The antimicrobial effects of Keggin-type HPAs on many bacteria and viruses have been investigated, but no information concerning the morphology and ultrastructure of fungal cells is available. The aim of this study was to evaluate the antifungal activity of Keggin-type Ag-HPA salts against *Sporothrix* spp. strains by analyzing the inhibition of growth and interference with melanin production and observing their effects on cell ultrastructure and membrane permeabilization.

## Materials and Methods

### Synthesis of HPA Salts

The acid forms of the HPAs H_3_[PW_12_O_40_], H_3_[PMo_12_O_40_], H_4_[SiW_12_O_40_], and H_6_[SiW_10_V_2_O_40_] were prepared by acidification with sulfuric acid and stoichiometric solutions of the proper oxoanions (Na_2_WO_4_•2H_2_O, Na_2_MoO_4_•2H_2_O or NaVO_3_•H_2_O and Na_2_SiO_3_•5H_2_O or Na_2_HPO_4_), followed by ether extraction and crystallization, as described elsewhere, with minor modifications [20]. The silver salts of the HPAs were prepared by simple precipitation with silver nitrate solution, and the precipitate obtained was washed and dried prior to use. The chosen HPA was dissolved to form a 1 × 10^-2^ mol l^-1^ solution, and to that an excess of 1 × 10^-2^ mol l^-1^ silver nitrate solution was added. The suspension formed was transferred to test tubes, and the precipitate was separated by centrifugation. The precipitate was then resuspended in sterile distilled water and centrifuged again. This step was repeated twice, and the final precipitate obtained was dried prior to use.

### Fourier-Transform Infrared Spectroscopy (FTIR)

The methodology was conducted according to previous work with minor modifications [20]. The FTIR spectra of the Ag-HPA salts were obtained from samples dispersed in KBr disks in the range of 4,000-400 cm^-1^ in a spectrophotometer model Prestige-21 (Shimadzu, Japan).

### X-Ray Diffraction

Ag-HPA salts (Ag_3_[PW_12_O_40_], Ag_3_[PMo_12_O_40_], Ag_6_[SiW_10_V_2_O_40_], and Ag_4_[SiW_12_O_40_]) were analyzed by X-ray diffraction using an X-ray diffractometer URD 65 model-SEIFERT/GE and an X-ray tube with a copper filament. Samples were treated previously at 90°C for 30 min, sieved through 200-mesh netting and maintained in a desiccator.

### Fungal Strains and Growth Conditions

Some selected strains present different levels of melanization, which, according to the literature, could have some influence on drug response mechanisms [21]. The strains used were *Sporothrix* spp. (CMDB/IOC 01980599, CMDB/IOC 01990699, CMDB/IOC 02050799, CMDB/IOC 02020699 and ATCC 32285) obtained from Instituto Oswaldo Cruz (IOC), Rio de Janeiro, Brazil. Our clinical strains were isolated from infected cats (LSASs01, LSASs02, LSASs03) by the clinical practitioner of the Veterinary Hospital of Universidade Estadual do Norte Fluminense Darcy Ribeiro, Campos dos Goytacazes, Rio de Janeiro, Brazil. Further, all samples were stored in plates with potato dextrose agar (PDA) (Himedia, India), which were supplemented with chloramphenicol (50 mg/ml) (Sigma-Aldrich, USA) and cycloheximide (400 mg/ml) (Sigma-Aldrich), and stored at 28°C. To obtain the yeast cells, new cultured cells were grown in Brain Heart Infusion (BHI) Agar (India) followed by incubation at 37°C.

### Reagents and Antifungals

The Ag-HPA salts were suspended in sterile distilled water to a ratio of 51.2 mg/ml. The antifungal drugs miconazole nitrate (Sigma-Aldrich), itraconazole and terbinafine (Sigma-Aldrich) were diluted in dimethyl sulfoxide (Sigma-Aldrich), and amphotericin B was diluted in sterile distilled water to obtain stock solutions of 12.8 mg/ml, which were maintained at -70°C. All reagents used as controls in this step were purchased from Sigma-Aldrich, USA.

### Growth Inhibition on *Sporothrix* spp.

The activity of the Ag-HPA salts on fungal growth was tested against three strains of *Sporothrix* spp. (ATCC 32285, CMDB/IOC 01980599 and LSASs01). To obtain yeast cell cultures, from each stock culture, a small fragment of agar containing colonies was transferred and spread onto Petri dishes containing Brain Heart Infusion Agar and allowed to grow at 37°C for 5 days. To access yeast forms, the isolates were maintained by transferring the cultures into the same medium weekly. A suspension of inoculum was obtained from yeast cells in a tube with 10 ml of sterile 0.85% saline, corresponding to 0.5 McFarland suspension of 5 × 10^5^ CFU/ml carried out in a photometer OD_550nm_ (Densimat, France), as established by the manufacturer. To assay the effect of each Ag-HPA salt (32 μg/ml) on yeast growth, 0.1 ml of fresh inoculum and 0.1 ml of each Ag-HPA salt were added to 1.8 ml BHI broth Infusion Broth (BHI) and incubated at 37°C. Optical density readings at 550 nm were taken at zero hour, and then readings were conducted every 24 h for the following 14 consecutive days. For cell growth, itraconazole (500 μg/ml), and medium plus HPA acid, and HCl (32 μg/ml) were used as positive and negative controls, respectively. The latter was used to evaluate the effect of H+ on fungal growth. The optical densities were plotted as a function of the incubation time for each fraction. Experiments were performed in triplicate and repeated two times. The inhibition rate was defined as the median value.

### Determination of MICs

Minimum inhibitory concentrations (MIC) for the Ag-HPA salts were determined using the broth microdilution technique, according to Clinical Laboratory Standards Institute M27-A3 recommendations [22]. The compound suspensions were twofold serially diluted in RPMI 1640 medium (India) buffered with 0.16 M MOPS (3-[N-morpholine] propane sulfonic acid) (Sigma-Aldrich) in 96-well tissue culture plates at concentrations varying from 512-0.125 μg/ml. Antifungals such as itraconazole, miconazole nitrate, terbinafine and amphotericin B (Sigma-Aldrich) were used as positive controls. An inoculum of yeast cells was obtained in saline solution (0.85%), corresponding to 0.5 McFarland suspension of 5 × 10^5^ CFU/ml, which was determined with a densitometer at OD_550nm_ and diluted 1:1000 in RPMI 1640 medium. Each well was filled with 0,1 ml of *Sporothrix* spp. inoculum and 0,1 ml of compound suspensions to obtain a final Ag-HPA salt concentration of 0.0625-256 μg/ml. All microtitration plates were incubated in a humid chamber at 37°C for 96 h. The MIC values were defined as the lowest concentration that imposed complete inhibition of *Sporothrix* spp. growth, which was revealed by adding 30 μl/well resazurin (100 μg/ml) reagent (Sigma-Aldrich). The tests were performed in triplicate on two different days.

### Interaction HPA-Antifungal Assay

To test for the interaction effect among HPA and antifungal drugs and further examine the synergistic-like effect of HPA and antifungal drugs on microorganisms, the Ag-HPA salts were tested together with the disks of antifungal agents such as itraconazole (ITC, 10 μg), amphotericin B (AMB, 100 μg), ketoconazole (KTC, 50 μg), fluconazole (FLC, 25 μg) and miconazole nitrate (MCZ, 50 μg). Ag-HPA salts were mixed with Brain Heart Infusion agar to obtain final concentrations of 128 and 256 μg/ml. To each Petri dish, 0,1 ml of yeast (CMDB/IOC 01980599 and clinical strain LSASs01) suspension, corresponding to 0.5 McFarland suspension of 5 × 10^5^ CFU/ml, which was determined with a densitometer at OD_550nm_ and spread by using a sterile swab. Antifungal disks were placed in the center of each Petri dish and incubated for 96 h at 37°C. The inhibition zone was measured with the aid of a digital caliper (Mitutoyo, Brazil) and compared with controls without HPA salt. The mycelial forms of CMDB/IOC 01980599 and the clinical strain LSASs01 were evaluated for synergistic-like effects after seven days in the presence of AMB (100 μg) and ITC (10 μg) individually and in combination with Ag-HPA salts at a final concentration of 128 μg/ml by the disk diffusion method in Potato Dextrose Agar. The conidia inoculum was prepared according to a previously reported procedure [23]. To each Petri dish, 0,1 ml of conidia suspension (0.5 McFarland) was spread by using a sterile swab. Disks were placed in the center of the Petri dishes and incubated for 192 h at room temperature (RT). Inhibition zones were measured and scored according to the manufacturer’s recommendations (CECON, Brazil).

To investigate whether the interaction between Ag-HPA salts and antifungal agents interfered with the melanization process of mycelial forms, the colonies were allowed to grow at RT in a dark environment. Colony melanization was photodocumented on days 15 and 30 of incubation.

### Membrane Permeabilization Assay

To investigate membrane permeabilization in fungi, a qualitative assay was used [24], with minor modifications, based on the uptake of SYTOX Green (Molecular Probes, Invitrogen, USA), an organic compound that fluoresces after interacting with nucleic acids in cells with compromised plasma membranes. The yeast cells (CMDB/IOC 01980599) were allowed to grow for 96 h in BHI broth in the presence or absence of the Ag-HPA salts (32 μg/ml), after which the solids were pelleted and washed in PBS, pH 7.0. A suspension (0,1 ml) of yeast cells was incubated with SYTOX Green (0.25 μM) for 30 min at 25°C and stirred at 100 rpm. The cells were observed under a light microscope (Axiophoto, Zeiss, Germany) equipped with DIC and an epifluorescence filter set for fluorescein detection (450-490 nm) at an emission wavelength of 500 nm. Itraconazole (500 μg/ml) was used as a positive control.

### Transmission Electron Microscopy (TEM) and Scanning Electron Microscopy (SEM)

For electron microscopy, the yeast cells (CMDB/IOC 01980599) grown for 96 h in Brain Heart Infusion Broth, in the presence or absence of 32 μg/ml Ag-HPA salts, were fixed at RT for 1 h in 2.5% glutaraldehyde (Sigma-Aldrich) in cacodylate buffer (Sigma-Aldrich) (0.1 M, pH 7.2) at RT.

For TEM, samples were postfixed using 1% osmium tetroxide (Sigma-Aldrich), diluted in potassium ferrocyanide (Sigma-Aldrich), dehydrated with a series of acetone and embedded in Epon resin Polybeded 812 (PolyScience). Ultrathin sections were stained with 2% uranyl acetate for 20 min and lead citrate for 5 min and examined using transmission electron microscopy (900-Zeiss Company, Germany) at an accelerating voltage of 80 keV. For SEM, samples treated as mentioned before were adhered to coverslips with poly-L-lysine, postfixed, dehydrated in ethanol, dried to critical point in CO_2_, and covered with gold for observation with a scanning electron microscope (912-Zeiss Company) at an accelerating voltage of 50 keV.

### Statistics

Data were analyzed with the Prism program. Statistical significances are shown as *p* < 0.05 values by Tukey’s test at the 5% level of significance.

## Results

### Synthesis

**Fig. 1:**
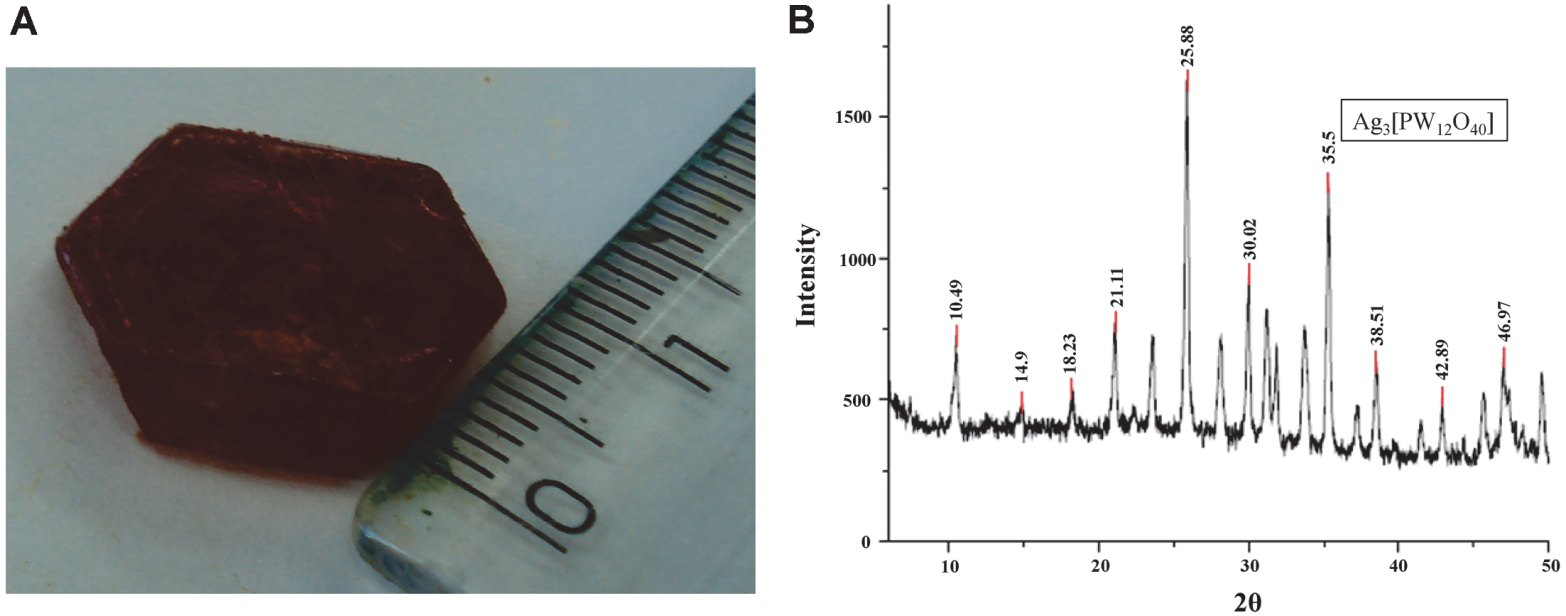
(A) Crystal of heteropolyacid H_6_[SiW_10_V_2_O_40_] obtained as large and brightly-colored crystals after two crystallization steps from water solutions. (B) Representative X-ray diffraction of Ag-HPA salt (Ag_3_[PW_12_O_40_]).

The heteropolyacids H_3_[PW_12_O_40_], H_3_[PMo_12_O_40_], H_4_[SiW_12_O_40_], and H_6_[SiW_10_V_2_O_40_] were obtained as large and brightly-colored crystals after two crystallization steps from water solutions, as shown in Fig. 1A for H_6_[SiW_10_V_2_O_40_]. The salts obtained were subjected to FTIR analysis, and their structures were compared with the literature data [20, 25]. The HPA salts obtained were silver dodecamolybdophosphate (Ag_3_[PMo_12_O_40_], silver dodecatungstophosphate (Ag_3_[PW_12_O_40_]), silver dodecatungstosilicate (Ag_4_[SiW_12_O_40_]) and silver decatungsto-vanadosilicate (Ag_6_[SiW_10_V_2_O_40_]). The obtained spectral results are summarized in Table 1.

### X-Ray Diffraction

According to the literature, no results for X-ray diffraction of HPA silver salts were found. However, the diffractogram shown in Fig. 1B is similar to the diffractogram of HPA H_3_[PW_12_O_40_] described previously [26], suggesting that the crystal structure was preserved upon substitution of H+ with Ag^+^ ions. The diffractograms were compared with X-ray diffractometer software on the PCPDFWIN database.

### Growth Inhibition Assay of HPAs on *Sporothrix* spp.

**Fig. 2:**
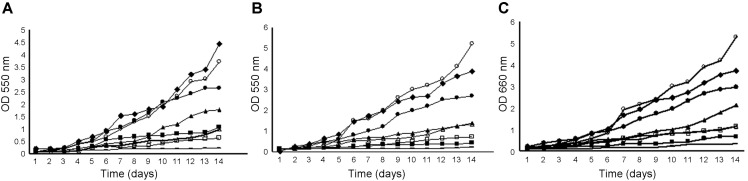
Effect of Ag-HPA salts on the growth of the yeast form of *Sporothrix*. (**A**) ATCC 32285 strain Growth curves; (**B**) CMDB/IOC 01980599 strain growth curve; (**C**) LSASs01 strain growth curve. Control (◆); Ag_3_[PMo_12_O_40_] ( ■ ); Ag_3_[PW_12_O_40_] ( □ ); Ag_6_[SiW_10_V_2_O_40_] ( ▲ ); Ag_4_[SiW_12_O_40_] (△); HPA acid ( ●), HCl (○) and itraconazole (**-**). The absorbance at 550 nm was used to measure growth.

In our experimental conditions, diverse effects on cell growth were observed depending on the fungal strain. The Ag-HPA salts were tested at a concentration of 32 μg/ml against three strains of *Sporothrix* spp. Toward strain ATCC 32285, the Ag-HPA salt Ag_3_[PMo_12_O_40_] showed the highest activity, with 93% inhibition of growth, followed by Ag_3_[PW_12_O_40_] (80%), and Ag_4_[SiW_12_O_40_] (68%) (Fig. 2A). Toward strain CMDB/IOC 01980599, Ag_3_[PW_12_O_40_] caused 84% inhibition, followed by Ag_3_[PMo_12_O_40_] and Ag_4_[SiW_12_O_40_], presenting 77% and 71%, respectively (Fig. 2B). Toward clinical strain LSASs01, the highest activity was demonstrated by Ag_3_[PMo_12_O_40_], which caused 83% inhibition, followed by Ag_4_[SiW_12_O_40_] (76%) and Ag_3_[PW_12_O_40_] (69%) (Fig. 2C). Of the Ag-HPA salts, Ag_6_[SiW_10_V_2_O_40_] inhibited cells the least at approximately 57% (ATCC 32285), 48% (CMDB/IOC 01980599) and 36% (LSASs01), respectively. The positive control in the presence of 500 μg/ml itraconazole presented total (100%) inhibition of growth against all strains tested. The negative controls, HPA acid in the form of H_3_[PMo_12_O_40_] and HCl presented a weak effect and were ineffective, respectively, on the growth of the strains tested when compared to the other treatments. (Figs. 2A-2C).

### Determination of MICs

The MICs for the salts of heteropolyacids with tungsten and molybdenum were tested against three clinical isolates of *Sporothrix* spp. and on the five standard strains as shown in Table 2. Among the Ag-HPA salts, the most expressive results were observed for the dodecamolybdophosphate of silver (Ag_3_[PMo_12_O_40_]) with MIC values varying from 8 to 16 μg/ml. The dodecatungstophosphate of silver (Ag_3_[PW_12_O_40_]) also presented expressive results with MIC values varying from 8 to 32 μg/ml. The MIC of dodecatungstosilicate of silver (Ag_4_[SiW_12_O_40_]) also varied from 16 to 32 μg/ml, and the MIC of divanadodecatungstosilicate of silver (Ag_6_[SiW_10_V_2_O_40_]) varied from 16 to 128 μg/ml. All antifungal drugs used as controls presented activity superior to Ag-HPA salts. Miconazole nitrate and terbinafine exhibited MIC values as low as 0.125-0.5 μg/ml and 0.5-2 μg/ml, respectively, which were even better than the MIC values of amphotericin B (1 to 8 μg/ml) and itraconazole (1 to 32 μg/ml), the recognized agents for the treatment of sporotrichosis (Table 2).

### Interactions of Ag-HPA Salts and Antifungal Drugs

Interactions among Ag-HPA salts and antifungal drugs were determined against CMDB/IOC 01980599 and LSASs01 clinical strains using the agar diffusion method. For some interactions between Ag-HPA salts and antifungal drugs, the inhibition zone results were significant but remained sensitive, as shown in Table 3. Table 3 (hatching) shows synergistic-like and antagonistic-like effects, as shown by the variation of Ag-HPA salts and antifungal combinations. Interactions of Ag-HPA salts and the antifungal drugs ITC and AMB changed their activity toward mycelial forms on both strains CMDB/IOC01980599 and clinical LSASs01, as shown in Table 4. The results show that for fungi treated with silver salts and further submitted to antifungal challenge, the activity between these two compounds against both mycelia and yeast cells remained synergistic-like when compared to untreated cells.

**Fig. 3:**
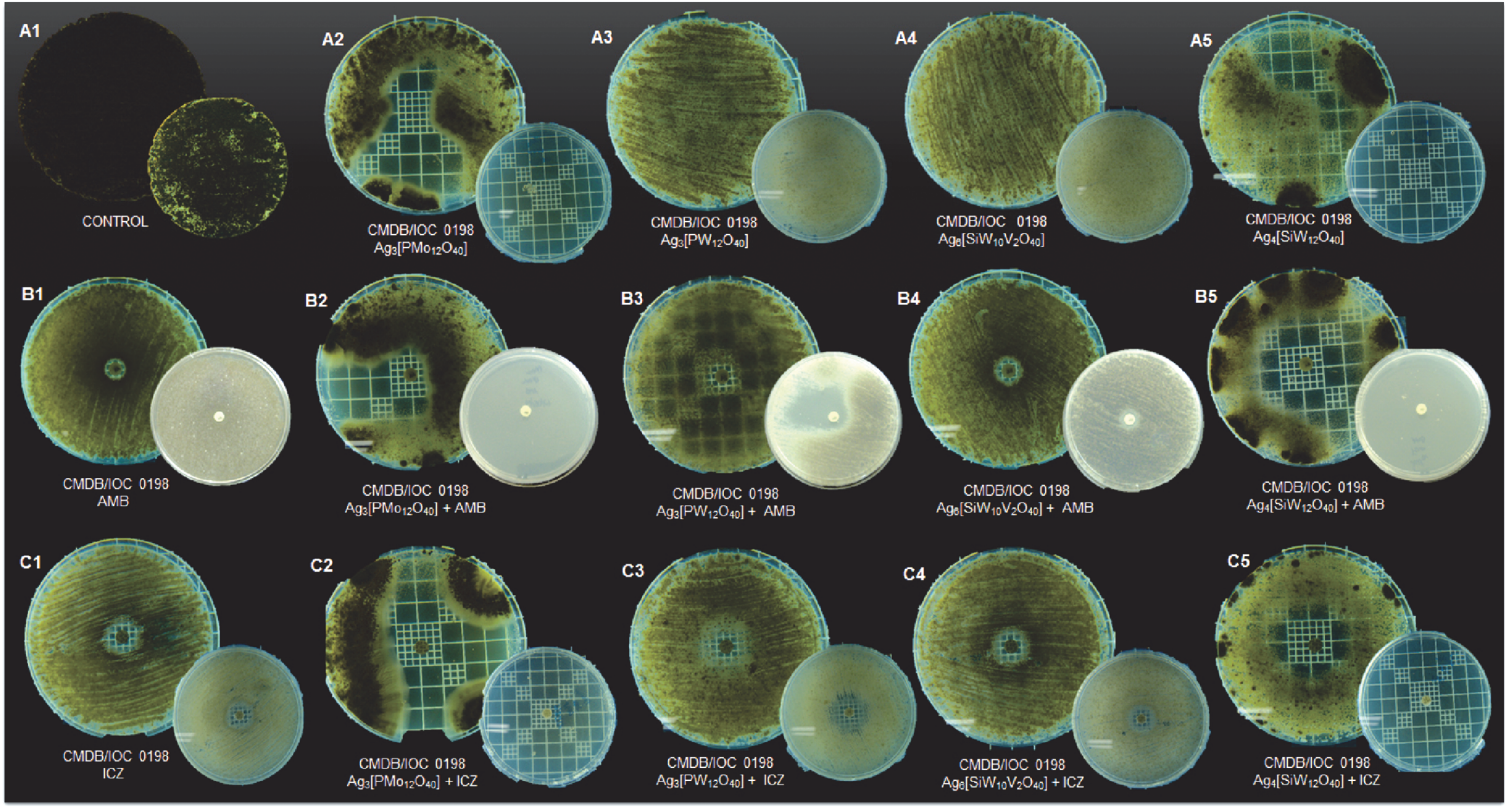
Sensibility, synergistic effects, and reduction in melanization of the mycelial form of the *Sporothrix* spp. strain CMDB/IOC 01980599 in the presence of Ag-HPA salts and antifungal drug disks by the diffusion agar method. (**A1**) Untreated control; (**A2-A5**) Growth in the presence of Ag-HPA salts Ag_3_[PMo_12_O_40_], Ag_3_[PW_12_O_40_], Ag_6_[SiW_10_V_2_O_40_], and Ag_4_[SiW_12_O_40_], respectively; (**B1**) Growth in the presence of amphotericin B (AMB); (**B2-B5**) Growth in the presence of combinations of AMB and Ag-HPA salts; (**C1**) Growth in the presence of itraconazole (ITC); (**C2-C5**) Growth in the presence of a combination of ITC and Ag-HPA salts. Figures correspond to 15 days (inferior small plates) and 30 days (superior big plates) of incubation.

**Fig. 4:**
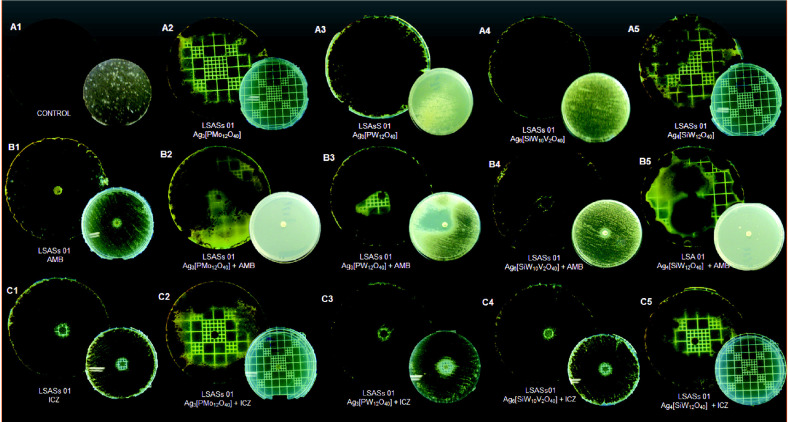
Sensibility, synergistic effects, and reduction in melanization of the mycelial form of the *Sporothrix* spp. clinical strain LSASs01 in the presence of Ag-HPA salts and antifungal drug disks by the diffusion agar method. (**A1**) Untreated control; (**A2-A5**) Growth in the presence of Ag-HPA salts Ag_3_[PMo_12_O_40_], Ag_3_[PW_12_O_40_], Ag_6_[SiW_10_V_2_O_40_], and Ag_4_[SiW_12_O_40_], respectively; (**B1**) Growth in the presence of amphotericin B (AMB); (**B2-B5**) Growth in the presence of combinations of AMB and Ag-HPA salts; (**C1**) Growth in the presence of itraconazole (ITC); (**C2-C5**) Growth in the presence of a combination of ITC and Ag-HPA salts. Figures correspond to 15 days (inferior small plates) and 30 days (superior big plates) of incubation.

The pattern of pigmentation of mycelial forms of both strains (CMDB/IOC 01980599 and clinical LSASs01) presented considerable alterations after Ag-HPA salts and the antifungal drugs ITC and AMB were combined, as shown in Figs. 3 and 4, suggesting interference in the melanization process. Only Ag_6_[SiW_10_V_2_O_40_] did not present a difference in pigmentation (Figs. 3C4 and 4C4) for either strain when compared to controls (Figs. 3A1 and 4A1).

### SYTOX Green Uptake Assay

**Fig. 5:**
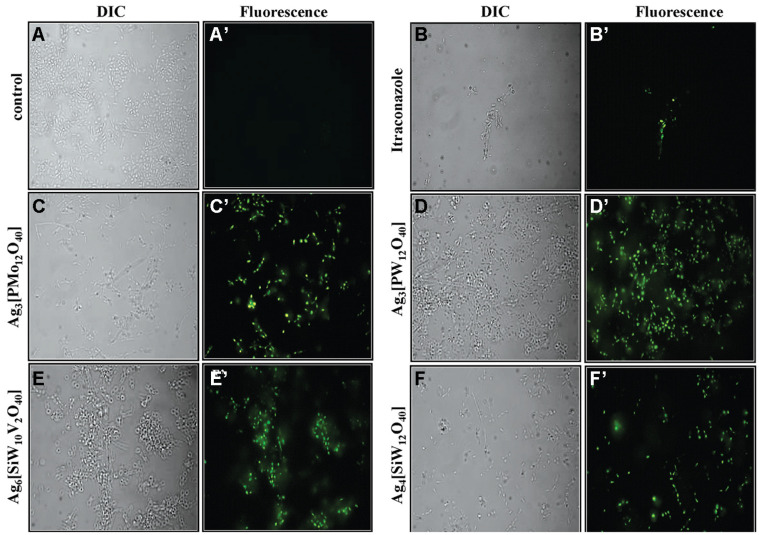
Membrane permeabilization assay performed by fluorescence microscopy of *Sporothrix* spp. yeast cells incubated with SYTOX Green. (**A** and **A’**) Control cells; (**B** and **B’**) cells treated with itraconazole (positive control); (**C** and **C’**) cells treated with Ag_3_[PMo_12_O_40_]; (**D** and **D’**) cells treated with Ag_3_[PW_12_O_40_], (**E** and **E’**) cells treated with Ag_6_[SiW_10_V_2_O_40_]; (**F** and **F’**) cells treated with Ag_4_[SiW_12_O_40_]. All conditions were viewed by DIC and by fluorescence. Magnification, 630X.

Membrane permeabilization of the yeast after 96 h incubation with Ag-HPA salts was observed on a light microscope by using vital dye staining. As shown in Fig. 5, no fluorescence was observed in the control test in the absence of Ag-HPA salt. For all treatments with Ag-HPA salts, fungal cells presented marked fluorescence due to the presence of SYTOX Green in their interior. Meanwhile, the panels with control and ITC-treated cells were clearly unchanged or less affected, respectively, when compared with the silver salt-treated cells.

### TEM Analysis

**Fig. 6:**
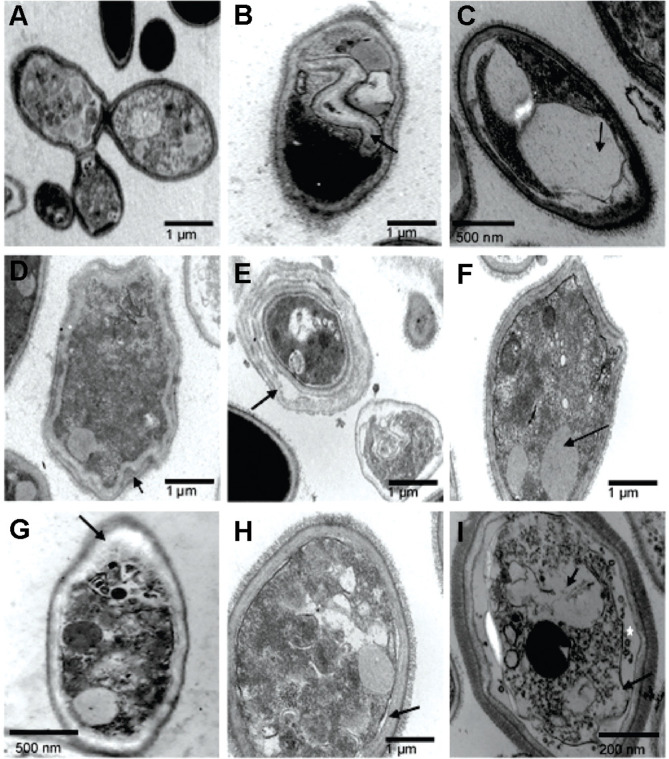
Activity of Ag-HPA salts on the internal structure of *Sporothrix* spp. yeast cells observed by TEM. (**A**) Control of cells in the absence of treatment showed a compact cell wall (CW) and dense microfibrillar material (FM); (**B, C**) Cells treated with Ag_3_[PMo_12_O_40_]; (**D, E, F**) Cells treated with Ag_3_[PW_12_O_40_]; (**G, H**) Cells treated with Ag_4_[SiW_12_O_40_]; (**I**) Cells treated with Ag_6_[SiW_10_V_2_O_40_]. Treatment showed several ultrastructural alterations, such as reduction in microfibrillar material (**B-E**), disruption of cell wall (**D, F**), large vacuolization (**B, C, G, I**), membrane detachment from the cell wall (**D, E, H, I**), and cell wall thickening (**B, D-G**).

For TEM images of control samples, budding cells presented intact cell walls, normal cytoplasmic density and dense microfibrillar material (Fig. 6A). In contrast, in the presence of Ag_3_[PMo_12_O_40_], yeast cells presented large abnormal vacuoles and less dense microfibrillar material (Figs. 6B and 6C). Treatment with Ag_3_[PW_12_O_40_] induced cell wall rupture and membrane detachment from the cell wall (Fig. 6D), cell wall thickness and detachment of the cell wall in many layers, which gave a “layer cake” aspect of the cell wall, and vacuolization and reduction of microfibrillar material (Figs. 6E-6F, black arrow). In the presence of Ag_4_[SiW_12_O_40_], cells presented cytoplasmic disorganization, cell wall thickening, loss of microfibrillar material (Fig. 6G, arrow) and membrane detachment from the cell wall (Fig. 6H, black arrow). In the case of Ag_6_[SiW_10_V_2_O_40_] treatment, yeast cells tended to present a more extreme membrane detachment (black arrow), leading to significant disorganization of membrane components, cytoplasm disorder, vacuolation, and the presence of vesicles in the space formed between the membrane and the vacuole (white star), despite the preserved external microfibrillar material (Fig. 6I, black arrow).

### Scanning Electron Microscopy – SEM

**Fig. 7:**
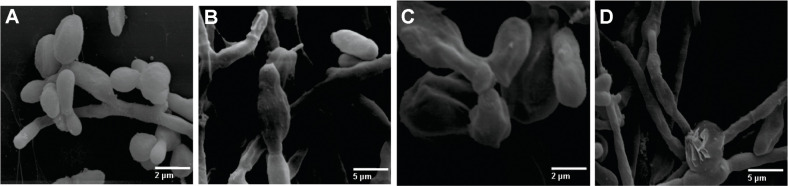
Activity of Ag-HPA salts on the surface structure of *Sporothrix* spp. yeast cells observed by SEM. (**A**) Control cells in the absence of treatment; (**B**) Cells treated with Ag_3_[PW_12_O_40_] showed hyphae-structure and slightly deformed conidia; (**C**) Cells treated with Ag_3_[PMo_12_O_40_] showed wrinkling of the conidia; (**D**) Cells treated with Ag_4_[SiW_12_O_40_] showed thinner hyphae-structures with abnormal budding compared to untreated yeast. Bars are in μm or nm.

SEM images of untreated yeast cells (control) showed a normal morphology with budding and regular surfaces (Fig. 7A). After treatment with Ag_3_[PW_12_O_40_] (Fig. 7B) or Ag_3_[PMo_12_O_40_] (Fig. 7C), cells presented a crimping surface. In the presence of Ag_4_[SiW_12_O_40_], cells presented many hyphae structures with little and few budding cells (Fig. 7C), in contrast to the smooth surface and a higher number of budding structures in control cells.

## Discussion

HPAs have been discussed as efficient, reusable, green and cost effective catalyst compounds that can be quickly synthesized [14, 27]. The present methodology used recrystallization, which has been used as the preferred method for the purification of HPAs [20]. The X-ray diffraction analysis (Fig. 1B) of Ag_3_[PW_12_O_40_] is representative of the Ag-HPA salt profile used in the present study. A similar diffractogram of the POM H_3_[PW_12_O_40_] was described [26] that resembles the results observed in this work but with different purposes and applications. Since 1972, the biological activities of POMs have been studied against tumor cells, viruses and bacteria [27]. A single report in the literature on the use of Keggin-type POMs against fungi was described by Curticãpean *et al.* [28], but the authors mentioned that it is only effective against bacteria. It has been known since ancient times that silver ions and silver salts can be used as antimicrobial agents because of their growth-inhibitory activity against microorganisms [29]. In this study, we examined the antifungal activity of four Keggin-type Ag-HPA salts, including one polyoxomolybdate (Ag_3_[PMo_12_O_40_]) and three polyoxotungstates (Ag_3_[PW_12_O_40_], Ag_4_[SiW_12_O_40_], and Ag_6_[SiW_10_V_2_O_40_]) on yeast and mycelial forms of *Sporothrix* spp., and their interactions with antifungal drugs associated with cell damage. The results presented in Figs. 3 and 4 were overwhelming by the noticeable observation of both the pigmentation and zone of inhibition differences of the treatments and antifungal drugs used.

The growth inhibition test and range of MICs of Ag-HPA salts against yeast *Sporothrix* spp. depended not only on the HPA but also on the yeast strain used. Both features were expected; the first was due to the varying physical-chemical properties of the HPA salts with different formulations. The second is well known and shown in the literature as fungi dimorphism, thermotolerance, cell wall components, and presence of melanin involved in the pathogenicity of *Sporothrix schenckii* [1, 30, 31]. The data showed that Ag-HPA salt phosphorus-containing heteroatoms and metals of the transition molybdenum (Ag_3_[PMo_12_O_40_]) and tungsten (Ag_3_[PW_12_O_40_]) presented a higher inhibition, with 78-93% and 69-85%, in the yeast form, respectively, compared with salt silicon-containing heteroatoms and metals tungsten (Ag_4_[SiW_12_O_40_]) and vanadium-substituent (Ag_6_[SiW_10_V_2_O_40_]), which inhibited yeast growth by 68-76% and 36-57%, respectively. In the MIC assay, P-containing Ag-HPA salts appeared to present overall lower MICs, although the values were higher than those presented by traditional antifungal agents (Table 2). Ag-HPA salt treatment revealed that the most resistant strain (ATCC 32285), which apparently did not present the ability to produce melanin pigment based on microscopy like the melanized clinical strains LSASs01, LSASs02 and LSASs03 (Table 2). In previous assays, strain ATCC 32285 showed pigmentation after growth in minimum medium with the addition of 1.0 M L-DOPA (3,4 dihydroxy-L-phenylalanine), a substrate for melanin synthesis (data not shown). This observation shows that culture conditions and the presence of exogenous substrate for pigment synthesis may favor melanization if the fungus possesses the enzymatic capacity. Previous work has shown that the addition of L-DOPA and glucose in the culture medium positively interferes with the melanization of *S. schenckii* and *Sporothrix brasiliensis* [1]. It was also demonstrated that melanin caused resistance to amphotericin B and to caspofungin against *Cryptococcus neoformans* by reducing the cell permeability, limiting the intracellular concentrations of antifungal drugs, or escaping from free radical components [32] and from freezing-thawing stress of clinical strain [33, 34].

When antimicrobial drugs that present only one mechanism of action are used, the high dosages required for efficacy often result in unwanted side effects and drug-resistance problems. Furthermore, to benefit from the effects of therapy, the synergy actions of antimicrobial agents may be evaluated to explore possible modes of action of new antibiotics or to generate antagonistic effects [35]. Using the agar diffusion method for synergism-like assays between antifungal drugs and Ag-HPA salts on yeast forms of *Sporothrix* spp., we also observed that their interactions depended on the strain tested. CMDB/IOC 010980599 and the clinical strain LSASs01 were sensitive to all antifungal drugs tested, except the last one, which presented intermediate sensitivity to ITC.

The antifungal activities of combinations of ITC with the Ag-HPA salts reported here revealed that P-based heteroatoms with (Ag_3_[PW_12_O_40_] and Ag_3_[PMo_12_O_40_]) were significantly more active against the macroscopic melanized clinical strain LSASs01. ITC and AMB were less active against the mycelial form of the CMDB/IOC 010980599 and LSASs01 strains when tested alone; however, when combined with Ag-HPA salts, their growth was retarded, and restricted pigmentation production suggested interference in melanization impairment after 15 days of incubation at room temperature. It is noteworthy to mention that ITC is a drug commonly used against this fungus in routine human and veterinary practice [7, 10]. Melanin may confer several vantages to fungi [36]; its production in *S. schenckii* was reported before, and the infection steps of this fungus were attributed to this component acting in a similar manner as *C. neoformans* [33]. Conversely, Si-based heteroatoms with Ag-HPA salts (Ag_4_[SiW_12_O_40_] and Ag_6_[SiW_10_V_2_O_40_]) were significantly more active against the CMDB/IOC 01980599 strain. Fungi also respond to stress stimuli by activating the SOS defense system by DNA repair, providing a survival mechanism [37]. In this matter, after contact of *Sporothrix* with Ag-HPA salts in the presence of some fungicide, resistant cells were rendered (Tables 3 and 4, and Fig. 4), suggesting a reaction of defense of the fungus or even pigment melanin interference. The combination of antifungal agents may be a successful strategy against fungal strains after developing resistance against traditional fungicides. An antagonistic-like effect was observed when all Ag-HPA salts were used in combination with FLC against both strains, suggesting that the contact of Ag-HPA with cell surface promoted a distinguished alteration within the *Sporothrix* cell. In the yeast *Candida albicans* the antagonistic interaction between FLC and silver nanoparticles (AgNP) was observed by others, and the authors attributed to release of silver ions that infiltrate into the yeast cells leading to the formation of NPs through reduction by organic compounds present in the cell wall and cytoplasm [47]. The LSASs01 strain also presented resistance in combination with some Ag-HPA salts and AMB, KTC or MCZ (Table 3).

The permeability of membranes of cells treated with Ag-salts has been described for some POMs. Kim et al. [38] inferred that Ag-NPs acted throughout the destruction of the membrane integrity of *Candida albicans,* and the antifungal activity of the Ag-NPs might be due to one of several intracellular components released during membrane disruption. Silver can also interact with the DNA of microorganisms, preventing cells from replicating [39]. This work observed that Ag-HPA salts in lower concentrations inhibit yeast *Sporothrix* spp., with MIC values ranging from 8 to 128 μg/ml. Data from fluorescence microscopy using SYTOX green dying suggest membrane permeabilization as one mode of action for all tested Ag-HPA salts on *Sporothrix* yeast cells (Fig. 5), which was verified by the number of dead cells treated with POMs compared to control cells treated with ITC, with a visible difference in the number of fluorescent cells. SYTOX green was used to demonstrate the antifungal activity of plant-derived antimicrobial peptides against *Saccharomyces cerevisiae* and pathogenic yeasts *C. albicans* and *Candida tropicalis* [24, 40].

Additionally, TEM analysis showed an interaction between Ag-HPA salts and the membrane structure and cytoplasm of the fungus. The ultrastructural alterations promoted by Ag-HPA salts showed modification of cell wall structure, cell vacuolization, cytoplasm disorder and membrane detachment. Overall, the normal cell wall of *S. schenckii* yeast presents an electron-transparent capsular or slime layer associated with electron-dense microfibrils [41]. In the present study, these microfibrils were preserved after treatment with Ag_6_[SiW_10_V_2_O_40_] (Fig. 6I), while the other treatments showed a clear reduction in microfibrillar material (Figs. 6B-6G). SEM images showed changes in the surface appearance of the cell surface from smooth to rough thus indicating outer cell wall damage with wrinkling after cells were exposed to Ag_3_[PW_12_O_40_], reflecting drastic internal ultrastructure changes, such as cell wall thickening and fraying (Figs. 6D and 6E). The larger surface area of silver compounds, providing better contact with microorganisms [42] and affecting yeast cells by attacking their membranes, thus disrupting the membrane potential [18], could explain the results presented in this work. Through TEM, other authors observed the formation of ‘pits’ on the membrane surfaces of *C. albicans,* leading to the formation of pores and culminating with cell death after silver treatment [18], and considerable accumulation of silver nanoparticles (AgNPs) outside the yeast, with a further releasing of silver ions into the cytoplasm of *C. albicans*, also observed by TEM [47]. Finally, pH may interfere with the presence of microorganisms at the site of infection, as well as enhance the activity of drugs during treatment [43]. It is likely that the moderate acidity of Ag-HPA salts and consequently the pH may favor their activity on *Sporothrix*, as evidenced in the data presented with vital dye and electronic microscopy.

Due to the increasing number of drug-resistant fungi, as well as the rise in immunocompromised patients, it has become of major importance to discover alternative antifungal substances to use against emerging mycoses such as sporotrichosis, notably in low-income and social index regions of developing countries such as Brazil [48, 49)], and against relevant mycotic diseases caused by other yeasts, such as *C. albicans* [50]. The Ag-HPA salts showed a consistent activity against *Sporothrix* spp. yeast cells causing growth inhibition and changes in cell structure, leading to their permeabilization, vacuolization and cytoplasmic disorders, in addition to pigmentation alterations with growth inhibition of their mycelial forms. The results herein strongly suggest that Keggin-type Ag-HPA salts are a promising class of compounds to be explored in the search for new antifungal drugs. The significance of this work relies on the first report concerning the effect of Ag-HPA salts against yeast cells of the pathogenic fungus *Sporothrix* spp.

## Figures and Tables

**Table 1:** FTIR spectrum main peaks assignment.

Literature*	Ag_3_[PW_12_O_40_]	Ag_3_[PMo_12_O_40_]	Ag_4_[SiW_12_O_40_]	Ag_6_[SiW_10_V_2_O_40_]	Assignment (cm^-1^)
1,055-1,100	1074	945	1109	1006	n P-O
900-1,000	892	896	938	909	n M-O
700-850	807	654	837	795	n M-O-M

*Tsigdinos, 1978

**Table 2:** In vitro antifungal activity of Ag-HPA salts and reference antifungals.

Strains *Sporothrix*									

Agente test		CMDB/IOC 01980599	CMDB/IOC 01990699	CMDB/IOC 02050799	CMDB/IOC 02020699	ATCC 32285	LSASs01	LSASs02	LSASs03
*Polyoxotungstates*									
Ag_3_[PW_12_O_40_]	[μg/ml]	8	8	32	32	16	32	32	32
	[nmol/ml]	22,5	2,5	9,9	9,9	4,9	9,9	9,9	9,9
Ag_6_[SiW_10_V_2_O_40_]	[μg/ml]	16	16	32	16	32	64	128	64
	[nmol/ml]	4,8	4,8	9,6	4,8	9,6	19,1	38,2	19,1
Ag_4_[SiW_12_O_40_]	[μg/ml]	16	32	16	16	16	16	16	16
	[nmol/ml]	4,8	9,6	4,8	4,8	4,8	4,8	4,8	4,8
*Polyoxomolybdate*									
Ag_3_[PMo_12_O_40_]	[μg/ml]	8	8	16	16	16	16	16	8
	[nmol/ml]	3,7	3,7	7,4	7,4	7,4	7,4	7,4	3,7
Antifungals									
ICZ	[μg/ml]	1	16	32	16	4	16	4	4
	[nmol/ml]	1,4	22,6	45,3	22,6	5,7	22,6	5,7	5,7
MCZ	[μg/ml]	0,125	0,125	0,5	0,5	0,25	0,5	0,5	0,5
	[nmol/ml]	0,3	0,3	1	1	0,5	1	1	1
TBF	[μg/ml]	0,5	0,5	2	0,5	0,5	0,5	0,5	0,5
	[nmol/ml]	1,5	1,5	6	1,5	1,5	1,5	1,5	1,5
AMB	[μg/ml]	2	1	4	4	1	8	8	4
	[nmol/ml]	2,16	1,1	4,3	4,3	1,1	8,6	8,6	4,3

**Table 3:** Inhibition zone values (in mm) showing synergism and antagonism between Ag-HPA salts at concentrations 128 and 256 μg/ml and commercial antifungal, by the disk diffusion assay on yeast form.

CMDB/IOC 01980299		Ag_3_[PW_12_O_40_]		Ag_3_[PMo_12_O_40_]		Ag_6_[SiW_10_V_2_O_40_]		Ag_4_[SiW_12_O_40_]	

Antifungal	Control	128 μ/ml	256 μ/ml	128 μ/ml	256 μ/ml	128 μ/ml	256 μ/ml	128 μ/ml	256 μ/ml
ITC	20 (S)e	22 (S)d	22 (S)d	22 (S)d	22 (S)d	25(S)c	25 (S)c	28 (S)b	32 (S)a
AMB	16 (S)d	20 (S)b	25 (S)a	16 (S)d	18 (S)c	16(S)d	21 (S)b	21 (S)b	25 (S)a
KTC	42 (S)d	48 (S)abc	49 (S)abc	52 (S)a	53 (S)a	45(S)d	50 (S)abc	48 (S)bc	51 (S)ab
FLC	20 (S)a	ND (R)d	6 (R)c	6 (R)c	8 (R)c	ND(R)d	6 (R)d	13 (R)b	15 (I)b
MCZ	45 (S)g	51 (S)e	54 (S)d	58 (S)c	63 (S)a	48(S)f	50 (S)e	56 (S)c	61 (S)b

**LSASs01**		**Ag_3_[PW_12_O_40_]**		**Ag_3_[PMo_12_O_40_]**		**Ag_6_[SiW_10_V_2_O_40_]**		**Ag_4_[SiW_12_O_40_]**	

**Antifungal**	**Control**	**128 μ/ml**	**256 μ/ml**	**128 μ/ml**	**256 μ/ml**	**128 μ/ml**	**256 μ/ml**	**128 μ/ml**	**256 μ/ml**

ITC	19 (I)e	30 (S)c	34 (S)b	36 (S)a	36 (S)a	21 (S)dc	21 (S)dc	21 (S)d	23 (S)d
AMB	22 (S)c	22 (S)bc	25 (S)a	15 (S)d	20 (S)c	11 (S)e	11 (S)e	11 (S)e	14 (S)d
KTC	62 (S)a	38 (S)de	40 (S)de	58 (S)bc	65 (S)b	32 (S)f	34 (S)f	36 (S)ef	40 (S)cd
FLC	40 (S)a	15 (I)c	26 (S)b	10 (R)d	11 (R)d	ND (R)e	ND (R)e	ND (R)e	ND (R)e
MCZ	41 (S)cde	39 (S)cde	43 (S)cd	53 (S)b	62(S)a	40 (S)de	40 (S)cd	44 (S)cd	49 (S)bc

S = Sensitive; R = Resistant; I = Intermediary; ND = not determined. Values of mean of inhibition zones in the same line, followed by identical lower case letters do not differ among themselves Tukey Test (*p* ≤ 0,05).

ITC ≥ 20 = Sensitive; ITC ≥ 12-19 = Intermediary; ITC ≤ 11 = Resistant;

AMB > 10 = Sensitive; AMB ≤ 10 = Intermediary or resistant;

KTC > 20= Sensitive; KET 10-20 = Intermediary; KET < 10 = Resistant;

FLC > 19= Sensitive; FLC 14-19 = Intermediary; FLC < 14 = Resistant;

MCZ > 20= Sensitive; MCZ 10-20 = Intermediary; MCZ < 10 = Resistant.

**Table 4:** Inhibition zone values (in mm) showing synergistic-like activity between Ag-HPA salts at concentration 128 μg/ml and commercial antifungal, by the disk diffusion assay on mycelia form.

CMBD/IOC 01980599		Ag_3_[PW_12_O_40_]	Ag_3_[PMo_12_O_40_]	Ag_6_[SiW_10_V_2_O_40_]	Ag_4_[SiW_12_O_40_]

Antifungal	Control	128 μg/ml	128 μg/ml	128 μg/ml	128 μg/ml
ITC	18 (I)d	30 (S)b	›70 (S)a	23 (S)c	›70 (S)a
AMB	8 (I)c	35 (S)b	›70 (S)a	9 (I)c	› 70 (S)a

**LSASs01**		**Ag_3_[PW_12_O_40_]**	**Ag_3_[PMo_12_O_40_]**	**Ag_6_[SiW_10_V_2_O_40_]**	**Ag_4_[SiW_12_O_40_]**

**Antifungal**	**Control**	**128 μg/ml**	**128 μg/ml**	**128 μg/ml**	**128 μg/ml**

ITC	18 (I)d	23 (S)b	›70 (S)a	20 (S)c	›70 (S)a
AMB	12 (S)c	32 (S)b	›70 (S)a	13 (S)c	›70 (S)a

S = Sensitive; R = Resistant; I = Intermediary; ND = not determined. Values of mean of inhibition zones in the same line, followed by identical lower case letters do not differ among themselves Tukey Test (*p* ≤ 0,05).

ITC ≥ 20 = Sensitive; ITC ≥ 12-19 = Intermediary; ITC ≤ 11= Resistant;

AMB > 10 = Sensitive; AMB ≤ 10= Intermediary or Resistant.
